# Urine lipoarabinomannan testing for diagnosis of pulmonary tuberculosis in children: a prospective study

**DOI:** 10.1016/S2214-109X(14)70195-0

**Published:** 2014-05

**Authors:** Mark P Nicol, Veronica Allen, Lesley Workman, Washiefa Isaacs, Jacinta Munro, Sandra Pienaar, Faye Black, Layla Adonis, Widaad Zemanay, Yonas Ghebrekristos, Heather J Zar

**Affiliations:** a Division of Medical Microbiology and Institute for Infectious Diseases and Molecular Medicine, University of Cape Town, Cape Town, South Africa; b Division of Clinical Pharmacology, Department of Medicine, University of Cape Town, Cape Town, South Africa; c Department of Paediatrics and Child Health, University of Cape Town, Cape Town, South Africa; d National Health Laboratory Service, Groote Schuur Hospital, Cape Town, South Africa

## Abstract

**Background:**

Urine tests for mycobacterial lipoarabinomannan might be useful for point-of-care diagnosis of tuberculosis in adults with advanced HIV infection, but have not been assessed in children. We assessed the accuracy of urine lipoarabinomannan testing for the diagnosis of pulmonary tuberculosis in HIV-positive and HIV-negative children.

**Methods:**

We prospectively recruited children (aged ≤15 years) who presented with suspected tuberculosis at a primary health-care clinic and paediatric referral hospital in South Africa, between March 1, 2009, and April 30, 2012. We assessed the diagnostic accuracy of urine lipoarabinomannan testing with lateral flow assay and ELISA, with mycobacterial culture of two induced sputum samples as the reference standard. Positive cultures were identified by acid-fast staining and tested to confirm *Mycobacterium tuberculosis* and establish susceptibility to rifampicin and isoniazid.

**Findings:**

535 children (median age 42·5 months, IQR 19·1–66·3) had urine and two induced specimens available for testing. 89 (17%) had culture-confirmed tuberculosis and 106 (20%) had HIV. The lateral flow lipoarabinomannan test showed poor accuracy against the reference standard, with sensitivity of 48·3% (95% CI 37·6–59·2), specificity of 60·8% (56·1–65·3), and an area under the receiver operating characteristic curve of 0·53 (0·46–0·60) for children without HIV and 0·64 (0·51–0·76) for children with HIV. ELISA had poor sensitivity in children without HIV (sensitivity 3·0%, 95% CI 0·4–10·5) and children with HIV (0%, 0·0–14·3); overall specificity was 95·7% (93·4–97·4).

**Interpretation:**

Urine lipoarabinomannan tests have insufficient sensitivity and specificity to diagnose HIV-positive and HIV-negative children with tuberculosis and should not be used in this patient population.

**Funding:**

US National Institutes of Health, the National Health Laboratory Services Research Trust, the Medical Research Council of South Africa, and the Wellcome Trust.

## Introduction

Microbiological confirmation of pulmonary tuberculosis in children is difficult. Collection of a sample from the lower respiratory tract is challenging because young children rarely spontaneously produce sputum. Sputum induction or gastric lavage are useful methods for obtaining respiratory samples,^1^ but require trained staff and basic equipment. Even when appropriate samples can be obtained, smear microscopy is rarely positive in children and mycobacterial culture is often required.^2^ The main drawback of culture is that treatment decisions often need to be made before results are available because the clinical course of tuberculosis can be rapid in children younger than 5 years. Moreover, culture requires advanced infrastructure and trained staff and is therefore seldom available in countries with the greatest burden of disease. We recently reported on the accuracy of Xpert MTB/RIF testing of induced sputum^3^ and nasopharyngeal aspirate^4^ specimens, which holds promise as a rapid and feasible alternative to culture in low-resource settings.

A urine test would simplify specimen collection for children with suspected pulmonary tuberculosis. Studies of urine tests^5^, ^6^, ^7^, ^8^, ^9^ for tuberculosis in adults with suspected tuberculosis have shown mixed results. Two approaches have been assessed. First, detection of small fragments of *Mycobacterium tuberculosis* DNA in urine^5^ showed initial promise, but early findings have not been confirmed. Second, urine tests for mycobacterial lipoarabinomannan, in both ELISA and lateral flow assay format, have been assessed in adults with suspected tuberculosis.^6^, ^7^, ^8^ The tests are sensitive in adults with advanced HIV disease but not in HIV-negative adults and HIV-positive adults with CD4 counts higher than 100 cells per L.^9^ The lateral flow assay version of the lipoarabinomannan test might be the first point-of-care test for tuberculosis with diagnostic utility. In a recent Comment in *The Lancet Global Health,*^10^ Van Rie outlined the need to assess the lipoarabinomannan test in children.

Young children with pulmonary tuberculosis develop disseminated disease more frequently than do adults; therefore, urine lipoarabinomannan testing might be useful for the diagnosis of pulmonary tuberculosis in children, including those without HIV. HIV-positive children are especially at risk for disseminated tuberculosis. Therefore, we prospectively assessed the accuracy of urine lipoarabinomannan testing in ELISA and lateral flow assay formats in HIV-positive and HIV-negative children presenting with suspected pulmonary tuberculosis to a primary health-care clinic or to a referral hospital in Cape Town, South Africa.

## Methods

### Study design and participants

We enrolled children aged 15 years or younger who presented with suspected pulmonary tuberculosis to Red Cross War Memorial Children's Hospital (a tertiary referral hospital) or Nolungile Clinic (a primary health-care facility), between March 1, 2009, and April 30, 2012. Suspected pulmonary tuberculosis was defined on the basis of cough of any duration and one of the following: household contact with an infectious tuberculosis source case within the preceding 3 months, loss of weight or failure to gain weight in the preceding 3 months (established by either documented weight loss on growth chart or parental report), a positive tuberculin skin test to purified protein derivative (2TU, PPD RT23, Staten Serum Institute, Denmark, Copenhagen), or a chest radiograph suggesting pulmonary tuberculosis (including airway compression or lymphadenopathy, diffuse miliary pattern, pleural effusion, or cavitary disease). A positive tuberculin skin test was defined as 5 mm or more of transverse induration in children with HIV or 10 mm or more in children without HIV. Children were excluded if they had received more than 72 h of treatment or prophylaxis for tuberculosis, if they were not resident in Cape Town and could not be followed up, if informed consent was not obtained, or if two induced sputum and one urine specimen could not be obtained.

Written, informed consent for enrolment in the study was obtained from a parent or legal guardian. The Research Ethics Committee of the Faculty of Health Sciences, University of Cape Town, and the Provincial Government of the Western Cape approved the study. Tuberculosis treatment was started at the discretion of the treating doctor on the basis of clinical, radiological, and microbiological information. Follow-up visits were done at 1, 3, and 6 months for children on tuberculosis treatment and at 1 and 3 months for those not treated. To assess response to treatment at follow-up, we recorded symptoms, signs, and weight, and repeated chest radiograph at completion of tuberculosis treatment.

### Procedures

A history and physical examination were done in all children by a study doctor. Routine clinical investigations included chest radiography, tuberculin skin test, and HIV testing in children with unknown HIV status (HIV rapid test, followed by a confirmatory PCR for children aged <18 months or HIV ELISA in children aged >18 months). CD4 count and HIV viral load were tested, and HIV-positive children were categorised with WHO clinical and immunological classification.^11^

Two consecutive induced sputum specimens were obtained in children for microbiological confirmation of tuberculosis as previously described^3^ and submitted for smear and liquid culture. Induced sputum specimens were transported within 2 h of collection to an accredited, centralised laboratory at Groote Schuur Hospital, Cape Town, and processed individually with standardised protocols by trained technologists. For both specimens, after decontamination with N-acetyl-L-cysteine and sodium hydroxide (1·0% final concentration), centrifuged deposits were resuspended in 1·5 mL phosphate buffer. A drop of induced sputum sediment was used for fluorescent acid-fast smear microscopy. Automated liquid culture testing (BACTEC MGIT, Becton Dickinson, Cockeysville, MD, USA) was done with 0·5 mL resuspended pellet. Cultures were incubated for up to 6 weeks. Positive cultures were identified by acid-fast staining followed by MTBDR*plus* testing (Hain Lifescience, Nehren, Germany) to confirm *M tuberculosis* and to establish susceptibility to rifampicin and isoniazid.

Urine was collected from children with specimen bags, unless children were old enough to voluntarily produce a specimen on demand. All urine specimens were frozen within 2 h of collection at −80°C, and were tested within 24 months of storage. For the lateral flow assay, 60 μL of thawed urine was applied to the test strip, incubated at room temperature for 25 min, visually inspected, and the intensity of any visualised test band was graded by comparison of band intensity with the manufacturer-supplied reference card by one research laboratory technician trained in this technique. Test band intensity was graded as zero if no band was visualised, and grade 1–5 for visualised bands, according to the band on the reference card that most closely matched the test band. Clearview tuberculosis ELISA (Alere, Waltham, MA, USA) was done according to the manufacturer's recommendations. Staff who did and recorded urine tests were masked to culture results and to the presenting clinical features. Results of lipoarabinomannan testing were not communicated to the treating doctor.

Tuberculosis diagnostic categorisation was based on clinical and microbiological investigations: definite tuberculosis (any induced sputum culture positive for *M tuberculosis*), not tuberculosis (culture negative, no tuberculosis treatment given, and documented resolution of symptoms and signs at 3 month follow-up visit), or possible tuberculosis (all other children, including children given tuberculosis treatment without culture confirmation and children with negative culture for *M tuberculosis* not given tuberculosis treatment and who did not have documented resolution of symptoms and signs at follow-up either because of loss to follow-up or persistent symptoms or signs at follow-up).

### Statistical analysis

For the primary analysis of the accuracy of lateral flow assay and ELISA, we used mycobacterial culture (ie, definite tuberculosis) as the reference standard. For a secondary analysis, we used a composite reference standard including a clinical decision to start tuberculosis treatment plus mycobacterial culture. We established sensitivity, specificity, and predictive values of the assays with 95% CIs. For lateral flow assay we derived receiver operating characteristic (ROC) curves by use of the different intensities of bands. Data were entered into Microsoft Access and analysed with STATA (version 10) and EpiInfo (version 6) for calculation of nutritional indices. We used simple descriptive statistics to characterise the study population. Normally distributed continuous data were summarised by mean and 95% CIs, and non-normally distributed continuous data by median and IQR. Categorical data were summarised as proportions with 95% CI. Statistical tests used were χ^2^ test of association, χ^2^ test for trend, Kruskal-Wallis, two-group test of equal proportions, and the ROC compare test.

We used multinomial regression to explore the possible effect of antiretroviral therapy (ART) and immune category on lateral flow assay (≥1 intensity) in children with HIV, and the possible effect of HIV status, age, tuberculin skin test result, and nutritional status on lateral flow assay (≥1 intensity) across the categories of tuberculosis diagnosis, with not tuberculosis as the reference category. Statistical tests were two-sided at α of 0·05.

### Role of funding source

The sponsor of the study had no role in study design, data collection, data analysis, data interpretation or writing of the report. The corresponding author had full access to all the data in the study and responsibility for the decision to submit for publication.

## Results

**Figure 1:**
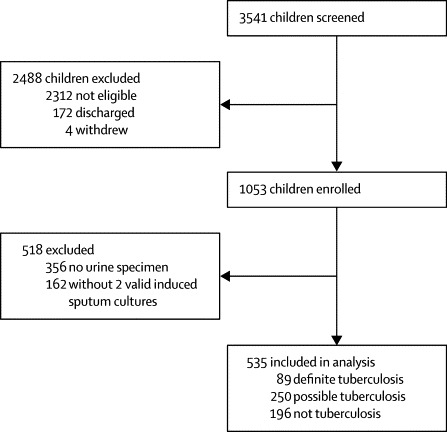
Study profile

We enrolled 1053 children, of whom 518 were excluded, mostly because no urine specimen was available (figure 1). We included 535 children with one urine and two induced sputum specimens in the analysis (201 [38%] from Nolungile Clinic and 334 [62%] from Red Cross War Memorial Children's Hospital), of whom 89 (17%) had definite tuberculosis, 250 (47%) had possible tuberculosis, and 196 (37%) did not have tuberculosis (figure 1). Of the 518 excluded children, the proportions with definite tuberculosis (63 [13%]), possible tuberculosis (281 [54%]), and not tuberculosis (173 [33%]) were similar to those included in the analysis.

Table 1 shows baseline characteristics of participants. Cough, fever, and weight loss were the most common presenting complaints (table 1). A clinical decision to start tuberculosis treatment was made in 294 of 535 (55%) children, of whom 87 of 89 were in the definite tuberculosis group and 207 of 250 were in the possible tuberculosis group. Children in the definite tuberculosis group not started on tuberculosis treatment were lost to follow-up after enrolment. 489 children had a Mantoux result (283 [58%] had a positive result). No child at Nolungile Clinic needed hospital admission and we recorded no in-hospital deaths in children enrolled at Red Cross War Memorial Children's Hospital. Median duration of hospital stay for children at Red Cross War Memorial Children's Hospital was 3 days (IQR 2–7). HIV infection was more common in children who presented to the hospital (84 of 334 [25%]) than in those at the primary care clinic (22 of 201 [11%]; odds ratio [OR] 2·73, 95% CI 1·64–4·53). 35 of 106 (33%) children with HIV were taking ART at enrolment. Of these children, 20 (57%) were started on tuberculosis treatment.

**Table 1:** Baseline characteristics of participants

		**Overall(n=535)**	**Definite tuberculosis (n=89)**	**Possible tuberculosis (n=250)**	**Not tuberculosis (n=196)**	p value*	**p value**†
Median age (years; IQR)		42·5 (19·1–66·3)	52·7 (26·8–93·3)	37·2 (17·8– 64·3)	42·3 (18·0–65·2)	0·007	0·005
Boys		302 (57%)	61 (69%)	140 (56%)	101 (52%)	0·03	0·007
Cough (any duration)		426 (81%)	76 (85%)	192 (77%)	158 (81%)	0·16	0·33
Fever‡		270 (51%)	45 (51%)	126 (50%)	99 (51%)	1·00	1·00
Weight loss§		334 (62%)	64 (72%)	160 (64%)	110 (56%)	0·03	0·01
HIV positive		106 (20%)	23 (26%)	49 (20%)	34 (17%)	0·25	0·10
Started on ART		35 (33%)	9 (39%)	11 (23%)	3 (44%)	0·16	0·71
WHO HIV clinical classification		106 (20%)	23 (26%)	49 (20%)	34 (17%)	..	0·01
	Stage 1	13 (12%)	0 (0%)	3 (6·1)	10 (29%)	0·001	..
	Stage 2	19 (18%)	1 (4%)	12 (24·5)	6 (18%)	0·12	..
	Stage 3	58 (55%)	15 (65%)	27 (55·1)	16 (47%)	0·40	..
	Stage 4	16 (15%)	7 (30%)	7 (14·3)	2 (6%)	0·05	..
WHO classification of HIV–associated immunodeficiency		99 (19%)	22 (25%)	48 (19%)	29 (15%)	..	0·78
	None or not significant	32 (32%)	8 (36%)	10 (21%)	14 (48%)	0·04	..
	Mild	14 (14%)	2 (9%)	10 (21%)	2 (7%)	0·21	..
	Advanced	12 (12%)	3 (14%)	7 (15%)	2 (7%)	0·67	..
	Severe	41 (41%)	9 (41%)	21 (44%)	11 (38%)	0·90	..
HAZ (IQR)		−1·0 (−2·1 to −0·1)	−1·1 (−2·2 to −0·2)	−1·1 (−2·1 to −0·1)	−0·9 (−1·9 to −0·1)	0·53	0·35
WAZ (IQR)		−0·9 (−1·9 to 0·1)	−1·1 −2·4 to −0·1)	−1·0 (−2·0 to −0·4)	−0·7 (−1·7 to 0·2)	0·07	0·61
WHZ (IQR)		−0·1 (−1·1 to 0·8)	−0·4 (−1·3 to 0·5)	−0·1 (−1·2 to 0·8)	0·0 (−0·8 to 0·9)	0·26	0·12
Underweight for age (WAZ ≤2)		121 (23%)	27 (30%)	56 (23%)	38 (20%)	0·16	0·04
TST		283 (58%)	65 (77%)	145 (63%)	73 (42%)	0·25	0·001

Data are n (%) unless otherwise stated. ART=antiretroviral therapy. HAZ=height-for-age *Z* score. WAZ=weight-for-age *Z* score. WHZ=weight-for-height *Z* score. TST=tuberculin skin test.

* Comparison between children with definite tuberculosis, possible tuberculosis, and not tuberculosis.

† Comparison between children with definite tuberculosis and children with not tuberculosis.

‡ Defined as core temperature >38·5°C.

§ Any documented weight loss on growth chart or by parental report.

Most children (317 [59%]) tested negative, or had a band intensity of 1 or stronger (111 [21%]) on lateral flow assay, with 52 (10%) having a band intensity of 2 or stronger, and the remaining 55 (10%) a band intensity of 3 or stronger. For the primary analysis, we assessed the accuracy of the lipoarabinomannan lateral flow assay with culture-confirmed tuberculosis as the reference standard. With 1 or greater band intensity as the cutoff, sensitivity and specificity were low in all participants (table 2). Accuracy was very poor for all band intensities, with an area under the ROC curve of 0·56 (95% CI 0·50–0·62; appendix).

**Table 2:** Sensitivity and specificity of lipoarabinomannan lateral flow assay and ELISA with culture-confirmed tuberculosis as the reference standard, stratified by HIV status and WHO HIV immunological staging

		**Sensitivity test positive/total positive (%, 95% CI)**	**Specificity test negative/total negative (%, 95% CI)**
**LFA band intensity ≥1**			
All children (n=535)		43/89 (48·3%, 37·6–59·2)	271/446 (60·8%, 56·1–65·3)
HIV positive (n=106)			
	All HIV positive	15/23 (65·2%, 42·7–83·6)	47/83 (56·6%, 45·3–67·5)
	None or not significant	6/8 (75·0%, 34·9 − 96·8)	13/24 (54·2%, 32·8–74·4)
	Mild	0/2 (0·0%, 0·0–65·8)	7/12 (58·3%, 27·7–84·8)
	Advanced	1/3 (33·3%, 0·8–90·6)	5/9 (55·6%, 21·2–86·3)
	Severe	7/9 (77·8%, 40·0–97·2)	17/32 (53·1%, 34·7–70·9)
HIV negative (n=429)		28/66 (42·4%, 30·3–55·2)	224/363 (61·7%, 56·5–66·7)
**LFA band intensity ≥2**			
All children (n=535)		26/89 (29·2%, 20·1–39·8)	365/446 (81·8%, 77·9–85·3)
HIV positive (n=106)			
	All HIV positive	10/23 (43·5%, 23·2–65·5)	66/83 (79·5%, 69·2–87·6)
	None or not significant	5/8 (62·5%, 24·5–91·5)	20/24 (83·3%, 62·6–95·3)
	Mild	0/2 (0·0%, 0·0–65·7)	9/12 (75·0%, 42·8–4·5)
	Advanced	1/3 (33·3%, 0·8–90·6)	5/9 (55·6%, 21·2–86·3)
	Severe	4/9 (44·4%, 13·7–78·8)	27/32 (84·4%, 67·2–94·7)
HIV negative (n=429)		16/66 (24·2%, 14·5–36·4)	299/363 (82·3%, 78·1–86·1)
**ELISA**			
All children (n=535)		2/89 (2·3%, 0·3–7·9)	427/446 (95·7%, 93·4–97·4
HIV positive (n=106)		0/23 (0·0%, 0·0–14·3)	79/83 (95·2%, 88·1– 98·7)
HIV negative (n=429)		2/66 (3·0%, 0·4–10·5)	348/363 (95·9%, 93·3–97·7)

LFA=lateral flow assay. ART=antiretroviral therapy.

**Figure 2:**
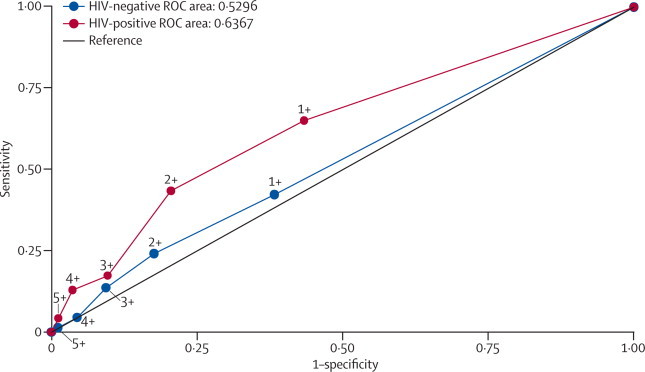
Receiver operating characteristic curve for lipoarabinomannan lateral flow assay with different band intensities (stratified by HIV status), with mycobacterial culture as the reference standard ROC=receiver operating characteristic.

The proportion of positive tests was similar in children with definite tuberculosis, possible tuberculosis, and not tuberculosis irrespective of band intensity cutoff (table 3). When accuracy was stratified by HIV status (figure 2), we recorded no significant difference between the area under the ROC curve for HIV-positive (0·64, 95% CI 0·51–0·76) and HIV-negative children (0·53, 0·46–0·60; p=0·14). When HIV-positive children were stratified by WHO immunological status, sensitivity was similar for children categorised as having no or insignificant immune suppression and those with severe immunosuppression; specificity remained poor, irrespective of amount of immune suppression (table 2).

**Table 3:** Children with a positive lipoarabinomannan lateral flow assay or ELISA, stratified by diagnostic categorisation

	**All children (n=535)**	**Definite tuberculosis (n=89)**	**Possible tuberculosis (n=250)**	**Not tuberculosis (n=196)**	p value*	**p value**†
LFA band intensity ≥1	218 (41%)	43 (48%)	95 (38%)	80 (41%)	0·24	0·24
LFA band intensity ≥2	107 (20%)	26 (29%)	42 (17%)	39 (20%)	0·04	0·08
ELISA positive	21 (44%)	2 (2%)	10 (4%)	9 (5%)	0·64	0·34

LFA=lateral flow assay.

* Comparison between children with definite tuberculosis, possible tuberculosis, and not tuberculosis.

† Comparison between children with definite tuberculosis and not tuberculosis.

Children with apparent false-positive lateral flow assay results (children in the not tuberculosis group with lateral flow assay ≥1 band intensity) were less likely than were other children to have a positive Mantoux test (23 of 68 [34%] *vs* 260 of 421 [62%]; relative risk [RR] 0·39, 95% CI 0·16–0·91), but were of a similar age to other children (median 35·4 months [95% CI 16·7–71·1] *vs* 44·3 months [21·1–66·3]).

Multinomial regression analysis showed that children older than 12 months with a positive Mantoux test had an increased risk of definite tuberculosis rather than not tuberculosis (RR 2·58, 95% CI 1·05–6·31 for age and 4·89, 2·69–8·87 for Mantoux status). After adjustment for the effect of age, HIV status, nutritional status, and Mantoux status, children with a positive lateral flow assay test were more likely than were children with a negative test result to have definite tuberculosis rather than not tuberculosis (RR 1·81, 95% CI 1·04–3·14). This effect was not noted when we compared children with possible tuberculosis with those without tuberculosis (1·11, 0·73–1·69).

In children with HIV, multinomial regression did not show any significant effect of ART or HIV immune category on the likelihood of children with a positive lateral flow assay result having definite tuberculosis rather than no tuberculosis (RR 1·60, 95% CI 0·52–5·01), or possible tuberculosis rather than no tuberculosis (0·65, 0·25–1·67).

Because culture is insensitive for the diagnosis of tuberculosis in children, we did a secondary analysis with a broad reference standard, which included both a clinical decision to start tuberculosis treatment and mycobacterial culture; however, accuracy was not improved (area under the ROC curve 0·50, 95% CI 0·45–0·54; appendix).

ELISA was positive in 21 of 535 (4%) children, including two of 89 (2%) with definite tuberculosis, ten of 250 (4%) with possible tuberculosis, and nine of 196 (5%) with not tuberculosis. With culture-confirmed tuberculosis as the reference standard, sensitivity was 2·3% (95% CI 0·3–7·9) and specificity 95·7% (93·4–97·4). We recorded no difference in sensitivity in HIV-positive and HIV-negative children (p=0·40; table 2), and this result was not affected by antiretroviral or tuberculosis treatment (data not shown).

**Figure 3:**
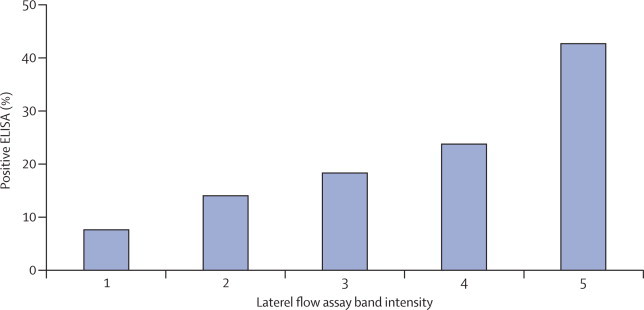
Children with a positive ELISA test, by lateral flow assay band intensity p<0·0001, test for trend.

We noted poor agreement between lipoarabinomannan lateral flow assay and ELISA for all lateral flow assay band intensity cutoff points (figure 3); however, we recorded a clear trend for increased likelihood of a positive ELISA test with increased intensity of bands on lateral flow assay (p<0·0001). Three of seven children (42·9%) with a lateral flow assay band intensity stronger than 5 had a positive ELISA test.

## Discussion

We report the first assessment of the accuracy of urine lipoarabinomannan testing with lateral flow assay and ELISA in children with suspected pulmonary tuberculosis (panel). Both assays did poorly against a reference standard of culture-confirmed tuberculosis. Because microbiological culture is insensitive for diagnosis of tuberculosis in children, we did a secondary analysis for the lateral flow assay, in which we included a clinical decision to start tuberculosis treatment in the reference standard; however, test accuracy did not improve. The proportion of children with a positive test (either lateral flow assay or ELISA) was similar in those with definite tuberculosis, possible tuberculosis, and not tuberculosis. By contrast with findings in adults with suspected tuberculosis,^13^, ^14^ accuracy of lateral flow assay was not substantially improved in children with HIV, and advanced immune suppression was not associated with increased sensitivity.

Panel **Research in context**

**Systematic review**

We searched PubMed for studies of lipoarabinomannan testing published in English from inception up to Jan 14, 2014, with the following search terms: “tuberculosis” and “diagnosis” and (“children” or “child” or “infant”) and (“lipoarabinomannan” or “clearview”). We did not identify any systematic reviews. We identified four narrative reviews and one primary research article^12^ that included lipoarabinomannan testing of 23 adolescents, but did not separately report on test accuracy in adolescents.

**Interpretation**

This is the first study of the accuracy of urine lipoarabinomannan testing in children to diagnose pulmonary tuberculosis, and showed poor sensitivity and specificity. Test accuracy was poor in children with HIV, although this study included only a few HIV-positive children with advanced immune suppression in whom test accuracy would be expected to be best. Further studies of this population of children are needed.

The reasons for the poor specificity of lipoarabinomannan testing are unclear. Most of the urine samples collected for this study were collected in urine bags, which are attached to the perineum. Bags might remain on the skin for several hours until the child produces urine; therefore, urine might become contaminated by bacteria from perineal skin or stool. The polyclonal antibodies used in these assays could have cross-reacted with contaminating bacteria, giving rise to false-positive results. Unfortunately we did not record the method of urine collection or do routine bacterial culture, and so cannot establish whether test performance was affected.

We recorded poor agreement between lateral flow assay and ELISA, with substantially fewer positive ELISA results than lateral flow assay. However, we noted that stronger band intensity on lateral flow assay was associated with an increased likelihood of a positive ELISA, suggesting that, at least in some cases, the same target was detected. This finding differs from studies done in adult patients that showed good correlation between lateral flow assay (with the ≥2 band intensity cutoff) and ELISA.^14^, ^15^ Poor agreement is unlikely to be attributable to technical reasons because our laboratory has previously done studies of adult patients that showed good agreement.^15^ Additionally, lateral flow assays were repeated with different batches of strips, with similar results (data not shown).

Although we hypothesised that lipoarabinomannan testing might show improved sensitivity in children without HIV compared with adults, this proved not to be the case. A possible explanation might be that both HIV-positive and HIV-negative children enrolled in this study did not have disseminated tuberculosis, but rather pulmonary tuberculosis, effectively confined to the respiratory system by the child's immune system. As evidence of this, none of the children were severely ill and needed intensive care support; none of the children recruited at Nolungile Clinic needed hospital admission, and no child recruited at Red Cross War Memorial Children's Hospital died in hospital. However, only a third of the children with HIV were taking ART, which would further enable effective containment of *M tuberculosis*. Paucity of ART treatment is worrying, highlighting gaps in the present ART programme, because all children with HIV should be receiving ART according to national policy.

Findings of studies^14^, ^15^ of HIV-positive adults have shown that lipoarabinomannan testing is useful in individuals with very advanced disease and severe immunosuppression (CD4 count <100 cells per μL); in these adults, testing has increased sensitivity and is useful for identification of adults at high risk of mortality.^16^ All children enrolled in our study were healthy, and all survived at least 6 months of follow-up and completion of tuberculosis treatment.

Graham and colleagues^17^ recently published a proposed consensus clinical case definition for intrathoracic tuberculosis in children. The categorisation used in this study was similar for patients with definite (or confirmed) tuberculosis. The definition used for not tuberculosis differs from the consensus definition in that we were able to follow up children, and hence accurately identify children who had recovered without treatment for tuberculosis. We chose not to further subcategorise children into possible and probable categories, but grouped these children together in one category to simplify the analysis in view of the poor performance of the assay in the definite and not tuberculosis groups.

Limitations of this study included a small sample size of children with HIV, especially those with advanced immune suppression, in whom assay sensitivity would be expected to be best. Therefore, we cannot make a definite conclusion for this group of children. However, the children enrolled in our study are representative of the population of South African children presenting to health-care facilities (both ambulatory and hospital) with suspected tuberculosis. Second, the poor sensitivity of mycobacterial culture in children could have resulted in an imperfect reference standard and cause spuriously low estimates of specificity. Although we tested two induced sputum samples, microbiological confirmation of tuberculosis was achieved in only a small proportion of all children for whom a clinical diagnosis of tuberculosis was made; this rate of culture positivity is similar to previously reported rates.^3^ However, the proportion of positive tests (lateral flow assay or ELISA) was similar in children with definite tuberculosis and not tuberculosis, with high diagnostic certainty. Therefore, the inability to confirm tuberculosis microbiologically is unlikely to have led to substantial underestimation of test specificity. Further, urine was frozen for up to 2 years before testing, which might have affected test performance; however, several large studies of adults, which showed promising sensitivity for lipoarabinomannan testing, also tested urine that had been previously frozen.^8^, ^14^

Sensitivity and specificity of urine lipoarabinomannan testing with both lateral flow assay and ELISA were poor in young children with suspected pulmonary tuberculosis. Therefore, these tests should not be used to diagnose tuberculosis in this patient population.
