# Supplementary material for: Urine lipoarabinomannan testing for diagnosis of pulmonary tuberculosis in children: a prospective study
Source: Lancet Glob Health. 2014 May;2(5):e278–84. doi: 10.1016/S2214-109X(14)70195-0 (PMC4012567; doi:10.1016/S2214-109X(14)70195-0)

THE LANCET Global Health

## Supplementary appendix

This appendix formed part of the original submission and has been peer reviewed. We post it as supplied by the authors.

Supplement to: Nicol MP, Allen V, Workman L, et al. Urine lipoarabinomannan testing for diagnosis of pulmonary tuberculosis in children: a prospective study. *Lancet Glob Health* 2014; published online April 8. http://dx.doi.org/10.1016/S2214-109X(14)70195-0.

Supplementary figure 1

Receiver operating characteristic curve for LAM lateral flow assay with different band intensities (all patients), using mycobacterial culture as the reference standard

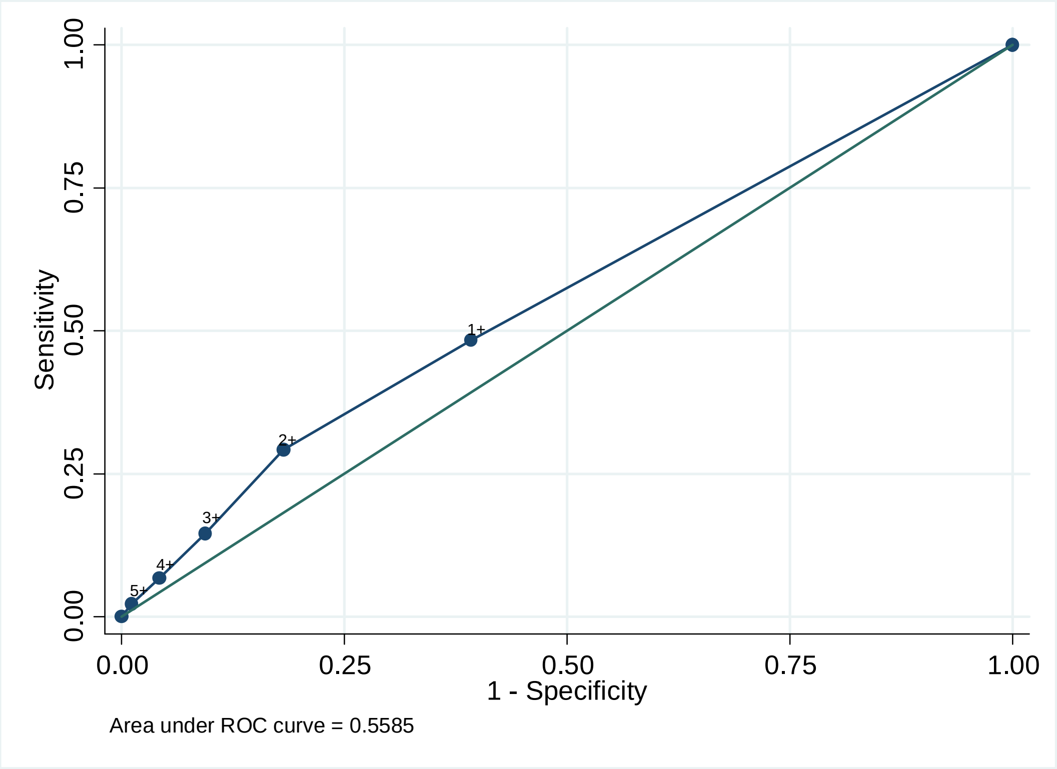

Supplementary figure 2

Receiver operating characteristic curve for LAM lateral flow assay with different band intensities (all patients), using a combined reference standard of a clinical decision to start tuberculosis therapy together with mycobacterial culture.

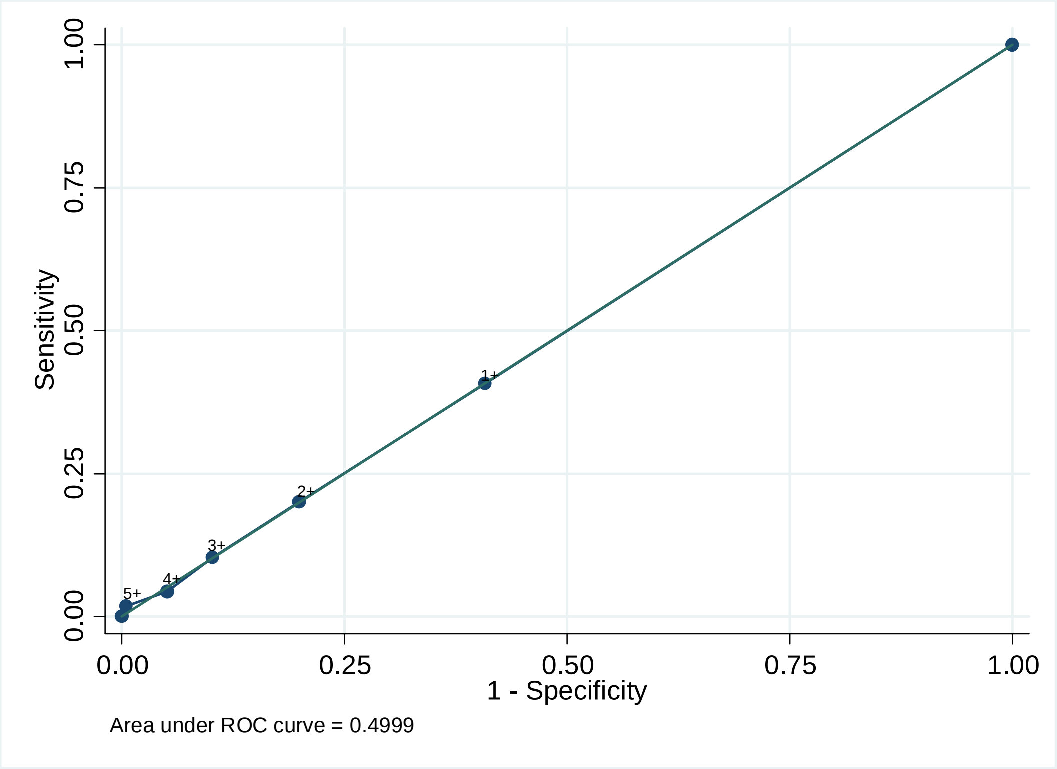

Supplement: Supplementary appendix [file mmc1.pdf]
